# Rates of compliance and adherence to high-intensity interval training in insufficiently active adults: a systematic review and meta-analysis protocol

**DOI:** 10.1186/s13643-020-01301-0

**Published:** 2020-03-17

**Authors:** Alexandre Santos, Chris Lonsdale, David Lubans, Diego Vasconcellos, Nathanial Kapsal, Mathew Vis-Dunbar, Mary E. Jung

**Affiliations:** 1 Faculty of Health and Social Development, University of British Columbia–Okanagan Campus, UCH106–1238 Discovery Avenue, Kelowna, BC V1V-1V9 Canada; 2 Institute for Positive Psychology & Education, Australian Catholic University, North Sydney, Australia; 3 School of Education, University of Newcastle Australia, Newcastle, Australia; 4 Library, University of British Columbia–Okanagan Campus, Kelowna, Canada

**Keywords:** Exercise, High-intensity interval training, Moderate-intensity Continuous training, Compliance, Adherence, Insufficiently Active adults, Systematic review, Meta-analysis

## Abstract

**Background:**

Both high-intensity interval training and moderate-intensity continuous training demonstrate beneficial physiological outcomes for active and insufficiently active populations. However, it remains unclear whether compliance to exercise in supervised settings translates to long-term adherence to physical activity in real-world, unstructured environments. To our knowledge, no comprehensive review is available on compliance and/or adherence rates to either modes of exercise for insufficiently active individuals. Furthermore, it is unclear which training modality insufficiently active individuals comply and/or adhere more readily to. Based on these gaps, the following two questions will be addressed: (1) What are compliance and adherence rates to high-intensity interval training for insufficiently active adults aged 18–65 years and (2) How do compliance and adherence rates differ between high-intensity interval training and moderate-intensity continuous training?

**Methods:**

Both observational and experimental studies that report on compliance and/or adherence rates to high-intensity interval training will be included. Relevant studies will be retrieved from Medline, EMBASE, PsychINFO, SPORTDiscus, CINAHL, and Web of Science using a pre-specified search strategy. Pre-defined inclusion and exclusion criteria will be used by two independent researchers to determine eligible studies. Of those meeting the inclusion criteria, data extraction and narrative synthesis will be completed, and where applicable, random-effects meta-analyses will be computed to compare compliance and adherence rates between high-intensity interval training and moderate-intensity continuous training. Meta-regressions and sensitivity analyses will be used to further explore factors that could influence aggregate effect sizes. Risk of bias will be assessed using established tools by the Cochrane association, and quality assessment of the cumulative evidence will be assessed using the GRADE approach.

**Discussion:**

Results from this study may have the potential to inform future physical activity recommendations and guidelines on the ideal mode of exercise for the general population. This review will add to the body of literature on the feasibility of high-intensity interval training for an insufficiently active population, conclusively addressing the ongoing debate of whether it is an appropriate exercise choice for this demographic. With this new information, individuals working towards a healthier lifestyle through physical activity engagement may be better equipped to make an evidence-based decision.

**Systematic review registration:**

This review has been registered in the PROSPERO database and assigned the identifier CRD42019103313.

## Background

Regular engagement in moderate-to-vigorous physical activity (MVPA) has been shown to decrease rates of various chronic diseases, including cardiovascular disease, diabetes mellitus, cancer, osteoporosis, obesity, hypertension, and depression [1]. Nonetheless, alarming rates of physical inactivity have been described as a global public health concern by the World Health Organization [2]. In response, physical activity interventions including continuous and intermittent aerobic exercise have been one of the main methods of attempting to increase physical activity levels in numerous populations. High-intensity interval training (HIIT) and moderate-intensity continuous training (MICT) are two types of aerobic exercises that have become increasingly popular in physical activity interventions. HIIT is known as an intermittent exercise protocol alternating between short bursts of high-intensity exercise (i.e., > 80% of maximum/peak heart rate) and recovery periods or light exercise [3]. In contrast, MICT entails a sustained effort of at least 10 min of exercise at a moderate intensity (i.e., between 64 and 75% of maximum/peak heart rate) [4].

Although both HIIT and MICT demonstrate beneficial physiological outcomes for active and insufficiently active populations (i.e., not meeting recommended physical activity guidelines) [5–10], both types of exercise have potential advantages and disadvantages. HIIT has been regarded as a time-efficient option that may elicit amplified physiological adaptations compared to MICT [3, 9]. Despite this, there is a debate as to whether HIIT is an appropriate training method for insufficiently active individuals due to questions of affective response, motivation levels, self-regulation, and adherence rates in this demographic [11–14]. The familiarity of MICT protocols may therefore serve as an advantage to exercise interventions targeting individuals not previously exposed to high-intensity exercise, as they may elicit more favourable affective responses to such protocols [11], consequently promoting continued engagement in physical activity. However, conflicting evidence exists [15] and more research is needed to determine whether HIIT is feasible for insufficiently active populations.

With its recent rise in popularity [16] and questions of whether HIIT can be adhered to long-term, the field is in need of a review to determine whether HIIT should be considered a feasible exercise modality. Where originally an abundance of research focused on the physiological benefits of HIIT and its comparison to more traditional forms of exercise such as MICT [i.e., 9], the pragmatic utility of such benefits can only be achieved if individuals are sustainably engaging in HIIT on their own. However, due to the field’s infancy and unstandardized methods of collecting real-world physical activity data, little attention has been given to compliance rates to such interventions and whether these interventions are indeed promoting an increase in regular long-term MVPA engagement, with one literature review of 14 interventions noting widely variable methods of reporting compliance and attrition [17].

Of interest to this study, a review by Weston and colleagues demonstrated that compliance to supervised HIIT or MICT programs, defined as the frequency of attendance to supervised exercise sessions, appear to be high for patients with lifestyle-induced cardiometabolic disease, with reported attendance rates to training sessions for nine studies ranging between 70 and 90% [3]. However, it remains unclear whether compliance to exercise in supervised settings translates to a long-term increase in physical activity adherence, defined as the number of minutes engaged in purposeful MVPA in unsupervised, unstructured environments. Although the results from Weston and colleagues’ review are promising, generalizability of findings to other populations that are insufficiently active is limited since only those with cardiometabolic disease were included. Therefore, it may be of value to include all individuals categorized as insufficiently active to be more representative of the general population. To our knowledge, no comprehensive review is available on compliance and/or adherence rates to either HIIT or MICT for all individuals categorized as insufficiently active. It is unclear whether insufficiently active individuals comply and/or adhere more readily to one training modality compared to the other, and whether such information may be helpful in guiding future practice for exercise prescription. Based on the gaps in the literature outlined, the following two questions will be addressed in this review:

1. What are compliance and adherence rates to HIIT for insufficiently active adults aged 18–65 years?
2. For studies that make direct comparisons, how do compliance and adherence rates differ between HIIT and MICT for insufficiently active adults aged 18–65 years?

## Methods

This protocol follows the Preferred Reporting Items for Systematic Reviews and Meta-Analysis–Protocol (PRISMA-P) guidelines [18]. Stages of study selection will be summarized using the PRISMA flow diagram (Additional file 1: PRISMA Flow Diagram). This review has been registered in the PROSPERO database and assigned the identifier CRD42019103313.

### Eligibility criteria

Eligibility of study inclusion in this systematic review will be based on pre-planned inclusion and exclusion criteria applied to each of the following domains: population, interventions, study type, and outcomes.

#### Population

The population eligible for inclusion consists of adults between the ages of 18 and 65 years classified as insufficiently active. In this review, insufficiently active adults are defined as those not meeting the current global physical activity guidelines of 150 min of MVPA per week for those between the ages of 18 and 65 years [10]. Adults identified as insufficiently active and having co-morbid disorders will also be included in this review. As part of the exclusion criteria, animal studies will not be eligible for inclusion and will be subsequently excluded during the title/abstract screening phase.

#### Interventions

Interventions must include HIIT, which has been previously defined by Weston and colleagues as alternating short bursts of high-intensity exercise with recovery periods or light exercise, where high intensity is measured as achieving a minimum of 80% maximum/peak heart rate [3]. Articles that make direct comparisons between compliance and adherence rates to HIIT and MICT in insufficiently active adults will be included in this review and subsequent meta-analyses in response to question 2. Consistent with the American College of Sports Medicine’s guidelines for exercise prescription, MICT is defined as achieving between 64 and 76% of maximum heart rate for a continuous period of at least 10 min [4].

#### Study type

Full-text, peer-reviewed articles of single-group observational studies, case studies/series, randomized controlled trials, pragmatic trials, multiphase optimization strategy trials, sequential multiple assignment randomized trials, and cross-over trials will be included in this review. Only full-text, peer-reviewed articles of randomized controlled trials, pragmatic trials, multiphase optimization strategy trials, sequential multiple assignment randomized trials, and cross-over trials will be included in meta-analyses. Qualitative studies, systematic reviews/meta-analyses, study protocols without a published study, grey literature, and published abstracts will be excluded from this review. The authors acknowledge that the exclusion of these types of studies may increase the chances of publication bias in this systematic review.

#### Outcomes

In addition to the aforementioned criteria, studies must include a quantifiable measure of either compliance to a supervised HIIT program or a measure of adherence to HIIT in unsupervised, unstructured environments, and where applicable, measures of compliance and/or adherence to MICT. For this review, compliance is defined as the frequency of attendance, in percentage, to exercise sessions of a HIIT program, and where applicable, MICT program. Compliance is measured at the end of a training protocol and may be accompanied by dropout rates and/or lost to follow-up rates. In contrast, adherence is defined as the number of minutes engaged in purposeful MVPA in unsupervised, unstructured environments after engaging in a HIIT program, and where applicable, a MICT program. For this review, self-report measures as well as wearable measures (i.e., heart rate monitors, accelerometers) of MVPA will be included.

### Data sources

The following databases will be searched for articles relevant to the research question: PubMed (Ovid), EMBASE (Ovid), PsychINFO (EBSCO), SPORTDiscus (EBSCO), CINAHL (EBSCO), and Web of Science. No date, sample, or language restrictions will be implemented in the search portion of this review. Further manual searches for articles will be conducted by checking the reference lists of each study deemed eligible for inclusion in this review, as well as expert consultation for relevant studies. Where possible and necessary, translations of eligible studies in a language other than English will be attempted.

If available, study protocols and trial registers of included studies will be sought as additional sources of information (i.e., for risk of bias assessments). In instances where not enough information is provided in included studies, personal communications with the study authors via the corresponding author will be carried out to request missing information.

The search strategy for this study will include main keywords of “high-intensity interval training,” “compliance,” and “adherence.” The full search strategy for this review can be found as an additional file (Additional file 2: Full Search Strategy). The search strategy has been pilot tested in each respective database and has been peer-reviewed by a chief librarian according to 2015 PRESS review guidelines [19].

### Study selection

Results of the full search strategy will be imported into the EndNote software [Version X9; Clarivate Analytics, 2018] for the manual removal of duplicates from the initial literature search. The articles that remain will be imported into the Covidence software [Veritas Health Innovation Ltd., 2015] for the screening and data extraction phases of this review.

All articles’ titles and abstracts will be screened by two independent researchers who will be following the pre-planned inclusion and exclusion criteria outlined in this document. Where there are discrepancies, the two researchers will convene and attempt to come to a consensus through review of the inclusion criteria and subsequent discussion. In situations when they cannot come to a consensus, a third independent researcher will screen the relevant studies to decide whether the inclusion criteria are met for each individual study. Similarly, full-text review will be completed by the two independent researchers to determine the final studies to be included. Where there are discrepancies, the same protocol used during title and abstract screening will be implemented.

Inclusion of studies in meta-analyses will be determined by the two independent researchers based on the inclusion criteria relevant to question 2 of this review. The inclusion of studies in meta-analyses will be determined after data extraction has occurred and will be dependent on the type of data reported in included studies (i.e., dichotomous versus continuous).

Manual searching of articles for inclusion in this review will be conducted by the two independent researchers. Sources of other potential articles will include articles found in previous systematic reviews that may be related to the topic of interest [i.e., 3] and individual expertise from the research team on articles in the field that may fit the inclusion criteria for this review but were not captured in the initial literature search.

### Data extraction

Data extraction will be divided between the two researchers responsible for the screening phase using a customized extraction form with the Qualtrics survey software [Version XM; Qualtrics International Inc., 2002]. An iterative process within the research team will be conducted to finalize the extraction form. The form will subsequently be piloted on five of the included studies, and modifications to the form will be made if needed, with post-hoc changes documented. Data will be sought for variables that fall within the following domains: author information, participant information, methods, interventions, primary, and secondary outcomes.

Variables that will be extracted from each study relating to author information will include title, publication year, authors, institutions and affiliations, sources of funding, and reported conflicts of interest.

Variables relating to participant information will include *n* values at each stage of the study, reasons for non-participation, dropout rates, lost to follow-up rates, treatment setting, eligibility criteria, definition of insufficiently active adult, and descriptive data including age, gender, ethnicity, socioeconomic status, diagnostic criteria, and treatment history.

Variables relating to methods will include study design, country, setting(s), methodological limitations reported, recruitment allocation, randomization process, data collection methods, blinding procedures, comparability of groups at baseline, and analysis methods.

Variables relating to interventions will include the number of groups, duration of treatment (number, frequency, and duration of exercise sessions), duration of follow-up, delivery method(s), and description of intervention(s).

Variables relating to primary and secondary outcomes will include descriptions of primary and secondary outcome measures, frequencies of measure implementation, and deviations and limitations reported relating to outcome measures. Main outcomes for which data will be sought include percentage of attendance to supervised exercise sessions, any other measure of compliance to exercise programs (i.e., achievement of exercise intensity), and number of minutes engaged in purposeful MVPA in real-world, unstructured environments at all available follow-up time points. Reasons for missing data will also be extracted. Additional outcomes that may influence compliance and/or adherence rates to HIIT or MICT (i.e., mediators/moderators) will also be extracted and will be agreed upon by the research team on an individual study basis. Such information will be used to inform pre-planned meta-regression analyses for this review.

### Risk of bias assessment

Once data extraction is completed, risk of bias will be assessed by two independent researchers. The Cochrane Risk of Bias Tool 2.0 [20] will be used to assess risk of bias in experimental studies, and if applicable, additional consideration will be given to cross-over trials according to Cochrane’s guidelines (*Handbook for Systematic Reviews of Interventions, Part 3, Section 16.4.3*). Quality of individual studies will not influence data synthesis, as studies will be considered for inclusion in meta-analyses regardless of risk of bias score.

The Risk of Bias in Non-Randomized Studies–Of Interventions (ROBINS-I) [21] tool will be used to assess risk of bias in included articles that do not meet the criteria of a randomized controlled trial.

Quality appraisal of the cumulative body of evidence will be assessed using the GRADE approach by two independent researchers. Primary outcomes will be assigned a quality of evidence GRADE score of either high, moderate, low, or very low quality based on the criteria outlined in the GRADE handbook [22]. Summary of findings tables, and evidence profile will be created using the GRADEpro Guideline Development Tool [23].

### Data synthesis and analysis

Data synthesis of included studies that are not experimental will be done using a narrative approach and using aggregate data of participants where possible. Results of experimental studies that compare compliance and/or adherence rates between HIIT and MICT will be quantitatively synthesized via random-effects meta-analyses for each primary outcome. Heterogeneity of articles will be analyzed post-hoc as opposed to the use of statistical heterogeneity measures such as Cochrane’s *Q* statistic and *I*-squared values due to substantial variability in interpretations of appropriate cutoff values [24]. If studies’ results are found to be heterogeneous, meta-regression analyses will be used to explore methodological moderators that may explain such heterogeneity. Meta-analyses will be conducted using the Comprehensive Meta-Analysis software for Windows. Mean values, change scores, and standard deviations will be used in the meta-analyses, and Hedge’s *g* will be used as measure of effect size.

#### Meta-regression analyses

Meta-regression analyses will be computed to determine whether specific factors moderate aggregate effect sizes calculated in meta-analyses. For both primary outcomes, meta-regression analyses will focus on the health status of participants (presence versus absence of disease), as previous research suggests a relationship between number/severity of co-morbid disease and levels of physical activity in other populations [25, 26]. For the primary outcome of adherence only, another meta-regression will be computed based on method of data measurement (self-report measure versus wearable measure), as some research has reported differences in energy expenditure estimates based on method of measurement [27, 28].

#### Sensitivity analyses

Sensitivity analysis will be performed to evaluate the robustness of meta-analysis results. Sensitivity analysis will be performed by excluding studies that are considered to be of high risk of bias, if any. The results of the meta-analyses would be considered reliable if sensitivity analysis does not significantly change the aggregate effect size calculations.

## Discussion

This systematic review will synthesize evidence from available literature regarding compliance to HIIT programs and determine whether HIIT is a feasible mode of exercise for insufficiently active adults in unsupervised, unstructured scenarios. Furthermore, the meta-analysis will help us determine whether insufficiently active adults are better able to comply and/or adhere to one mode of exercise over another. This study will add to the body of literature on the feasibility of HIIT for an insufficiently active population, conclusively addressing the ongoing debate of whether HIIT is an appropriate choice for this demographic. With this new information, individuals working towards a healthier lifestyle through physical activity engagement may be better equipped to make an evidence-based decision.

## Supplementary information

**Additional file 1:.** PRISMA Flow Diagram. Diagram to be populated with study selection process according to PRISMA guidelines.

**Additional file 2:.** Full Search Strategy. List of all search terms for each database included in the systematic review.

**Additional file 3:.** PRISMA-P Checklist. Completed PRISMA-P checklist related to submitted protocol.

## Data Availability

The datasets used and/or analyzed during the current study are available from the corresponding author on reasonable request.

## Abbreviations

HIIT: High-intensity interval training
MICT: Moderate-intensity continuous training
MVPA: Moderate-to-vigorous physical activity
PRISMA-P: Preferred Reporting Items for Systematic Reviews and Meta-Analysis–Protocol
ROBINS-I: Risk of Bias in Non-Randomized Studies–Of Interventions
