# Supplementary figures and images for: Rates of compliance and adherence to high-intensity interval training in insufficiently active adults: a systematic review and meta-analysis protocol
Source: Syst Rev. 2020 Mar 17;9:56. doi: 10.1186/s13643-020-01301-0 (PMC7077158; doi:10.1186/s13643-020-01301-0)

## PRISMA Flow Diagram – Adherence to HIIT

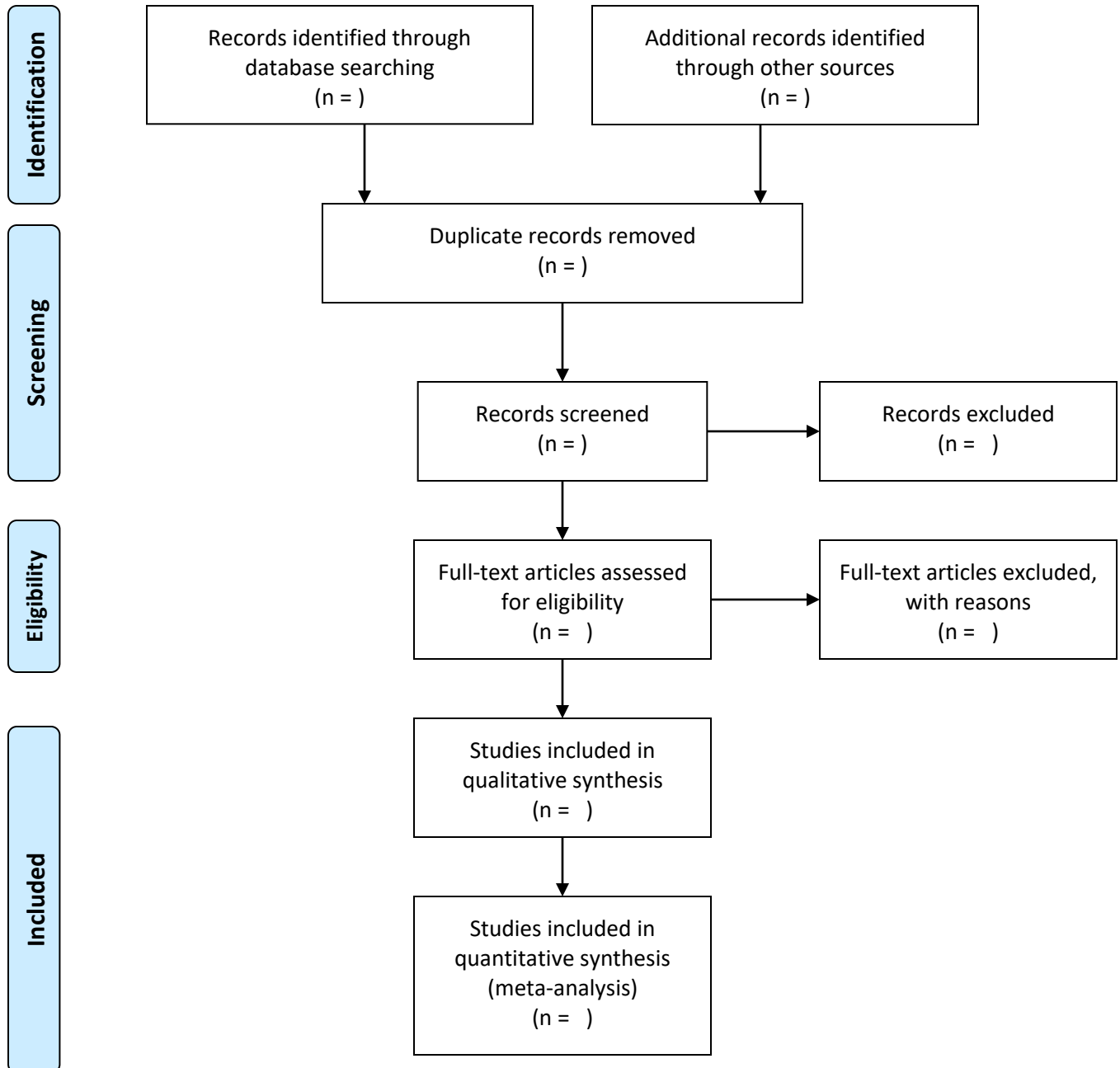

Supplement: Supplementary file 1 — Additional file 1:. PRISMA Flow Diagram. Diagram to be populated with study selection process according to PRISMA guidelines. [file 13643_2020_1301_MOESM1_ESM.pdf]
