# Supplementary material for: Rates of compliance and adherence to high-intensity interval training in insufficiently active adults: a systematic review and meta-analysis protocol
Source: Syst Rev. 2020 Mar 17;9:56. doi: 10.1186/s13643-020-01301-0 (PMC7077158; doi:10.1186/s13643-020-01301-0)
Supplement: Supplementary file 2 — Additional file 2:. Full Search Strategy. List of all search terms for each database included in the systematic review. [file 13643_2020_1301_MOESM2_ESM.pdf]

# Rates of compliance and adherence to high-intensity interval training in inactive adults: A systematic review and meta-analysis

Alexandre Santos
Chris Lonsdale
David Lubans
Diego Vasconcellos
Nathanial Kapsal
Mary Jung

## Full Search Strategy

Databases:

- Medline
- EMBASE
- PsychINFO
- SPORTDiscus
- CINAHL
- Web of Science

Date range:

- All dates included.

Languages:

- All languages included (where applicable, attempts at translation will be conducted).

Population:

- All populations included (to be specified in inclusion/exclusion criteria during screening).

Study Type:

- All study types included (to be specified in inclusion/exclusion criteria during screening).

Medline (Ovid) Legend:

- .mp = search term is found within either the title, abstract, or author-supplied keywords of the article.
- / = MeSH heading found in MeSH database.

("high intensity" adj3 train*).mp. OR
("high intensity" adj3 exercis*).mp. OR
("aerobic interval train*").mp. OR
("aerobic circuit train*").mp. OR
("aerobic interval exercis*").mp. OR
("aerobic intermittent exercis*").mp. OR
("aerobic circuit exercis*").mp. OR
("interval train*").mp. OR
("intermittent train*").mp. OR
("circuit train*").mp. OR
("interval exercis*").mp. OR
("intermittent exercis*").mp. OR
("circuit adj2 exercis*").mp. OR
(sprint adj2 train*).mp. OR
(sprint adj2 exercis*).mp. OR
(HIIT).mp. OR
(exp circuit-based exercise/) OR
(exp high-intensity interval training/)

AND

(adher*).mp. OR
(complian*).mp. OR
(participat*).mp. OR
(dropout).mp. OR
(complet*).mp. OR
(retention).mp. OR
(attend*).mp. OR
("lack of uptake").mp. OR
(frequen*).mp. OR
("lost to follow-up").mp. OR
("attendance frequen*").mp. OR
(exp treatment adherence and compliance/) OR
(exp lost to follow-up/) OR
(exp surveys and questionnaires/) OR

EMBASE (Ovid) Legend:

- .mp = search term is found within either the title, abstract, or author-supplied keywords of the article.
- / = Subject heading in Emtree database

("high intensity" adj3 train*).mp. OR
("high intensity" adj3 exercis*).mp. OR
("aerobic interval train*").mp. OR
("aerobic circuit train*").mp. OR
("aerobic interval exercis*").mp. OR
("aerobic intermittent exercis*").mp. OR
("aerobic circuit exercis*").mp. OR
("interval train*").mp. OR
("intermittent train*").mp. OR
("circuit train*").mp. OR
("interval exercis*").mp. OR
("intermittent exercis*").mp. OR
("circuit adj2 exercis*").mp. OR
(sprint adj2 train*).mp. OR
(sprint adj2 exercis*).mp. OR
(HIIT).mp. OR
(exp circuit training/) OR
(exp high-intensity interval training/)

AND

(adher*).mp. OR
(complian*).mp. OR
(participat*).mp. OR
(dropout).mp. OR
(complet*).mp. OR
(retention).mp. OR
(attend*).mp. OR
("lack of uptake").mp. OR
(frequen*).mp. OR
("lost to follow-up").mp. OR
("attendance frequen*").mp. OR
(exp patient compliance/) OR
(exp follow up/) OR
(exp questionnaire/) OR

PsychINFO (EBSCO) Legend:

- AB = Search term is found within abstract of the article.
- TI = Search term is found within the title of the article.
- DE = Subject heading in PsychINFO thesaurus.

TI ("high intensity" N3 train*) OR
TI ("high intensity" N3 exercis*) OR
TI ("aerobic interval train*") OR
TI ("aerobic circuit train*") OR
TI ("aerobic interval exercis*") OR
TI ("aerobic intermittent exercis*") OR
TI ("aerobic circuit exercis*") OR
TI ("interval train*") OR
TI ("intermittent train*") OR
TI ("circuit train*") OR
TI ("interval exercis*") OR
TI ("intermittent exercis*") OR
TI ("circuit N2 exercis*") OR
TI (sprint N2 train*) OR
TI (sprint N2 exercis*) OR
TI (HIIT) OR
AB ("high intensity" N3 train*) OR
AB ("high intensity" N3 exercis*) OR
AB ("aerobic interval train*") OR
AB ("aerobic circuit train*") OR
AB ("aerobic interval exercis*") OR
AB ("aerobic intermittent exercis*") OR
AB ("aerobic circuit exercis*") OR
AB ("interval train*") OR
AB ("intermittent train*") OR
AB ("circuit train*") OR
AB ("interval exercis*") OR
AB ("intermittent exercis*") OR
AB ("circuit N2 exercis*") OR
AB (sprint N2 train*) OR
AB (sprint N2 exercis*) OR
AB (HIIT)

AND

TI (adher*) OR
TI (complian*) OR
TI (participat*) OR
TI (dropout) OR
TI (complet*) OR
TI (retention) OR
TI (attend*) OR
TI ("lack of uptake") OR
TI (frequen*) OR

TI ("lost to follow-up") OR
TI ("attendance frequen*") OR
AB (adher*) OR
AB (complian*) OR
AB (participat*) OR
AB (dropout) OR
AB (complet*) OR
AB (retention) OR
AB (attend*) OR
AB ("lack of uptake") OR
AB (frequen*) OR
AB ("lost to follow-up") OR
AB ("attendance frequen*") OR
DE (compliance) OR
DE (treatment compliance) OR
DE (dropouts) OR
DE (potential dropouts) OR
DE (treatment dropouts) OR
DE (measurement)

SPORTDiscus (EBSCO) Legend:

- AB = Search term is found within abstract of the article.
- TI = Search term is found within the title of the article.
- DE = Subject heading in SPORTDiscus thesaurus.

TI ("high intensity" N3 train*) OR
TI ("high intensity" N3 exercis*) OR
TI ("aerobic interval train*") OR
TI ("aerobic circuit train*") OR
TI ("aerobic interval exercis*") OR
TI ("aerobic intermittent exercis*") OR
TI ("aerobic circuit exercis*") OR
TI ("interval train*") OR
TI ("intermittent train*") OR
TI ("circuit train*") OR
TI ("interval exercis*") OR
TI ("intermittent exercis*") OR
TI ("circuit N2 exercis*") OR
TI (sprint N2 train*) OR
TI (sprint N2 exercis*) OR
TI (HIIT) OR
AB ("high intensity" N3 train*) OR
AB ("high intensity" N3 exercis*) OR
AB ("aerobic interval train*") OR
AB ("aerobic circuit train*") OR
AB ("aerobic interval exercis*") OR
AB ("aerobic intermittent exercis*") OR
AB ("aerobic circuit exercis*") OR
AB ("interval train*") OR
AB ("intermittent train*") OR
AB ("circuit train*") OR
AB ("interval exercis*") OR
AB ("intermittent exercis*") OR
AB ("circuit N2 exercis*") OR
AB (sprint N2 train*) OR
AB (sprint N2 exercis*) OR
AB (HIIT) OR
DE (interval training) OR
DE (high-intensity interval training) OR
DE (circuit training)

AND

TI (adher*) OR
TI (complian*) OR
TI (participat*) OR
TI (dropout) OR

TI (complet*) OR
TI (retention) OR
TI (attend*) OR
TI ("lack of uptake") OR
TI (frequen*) OR
TI ("lost to follow-up") OR
TI ("attendance frequen*") OR
AB (adher*) OR
AB (complian*) OR
AB (participat*) OR
AB (dropout) OR
AB (complet*) OR
AB (retention) OR
AB (attend*) OR
AB ("lack of uptake") OR
AB (frequen*) OR
AB ("lost to follow-up") OR
AB ("attendance frequen*") OR
DE (exercise adherence) OR
DE (treatment effectiveness) OR
DE (health status indicators) OR
DE (health surveys)

CINAHL (EBSCO) Legend:

- AB = Search term is found within abstract of the article.
- TI = Search term is found within the title of the article.
- MH = Subject heading in CINAHL headings.

TI ("high intensity" N3 train*) OR
TI ("high intensity" N3 exercis*) OR
TI ("aerobic interval train*") OR
TI ("aerobic circuit train*") OR
TI ("aerobic interval exercis*") OR
TI ("aerobic intermittent exercis*") OR
TI ("aerobic circuit exercis*") OR
TI ("interval train*") OR
TI ("intermittent train*") OR
TI ("circuit train*") OR
TI ("interval exercis*") OR
TI ("intermittent exercis*") OR
TI ("circuit N2 exercis*") OR
TI (sprint N2 train*) OR
TI (sprint N2 exercis*) OR
TI (HIIT) OR
AB ("high intensity" N3 train*) OR
AB ("high intensity" N3 exercis*) OR
AB ("aerobic interval train*") OR
AB ("aerobic circuit train*") OR
AB ("aerobic interval exercis*") OR
AB ("aerobic intermittent exercis*") OR
AB ("aerobic circuit exercis*") OR
AB ("interval train*") OR
AB ("intermittent train*") OR
AB ("circuit train*") OR
AB ("interval exercis*") OR
AB ("intermittent exercis*") OR
AB ("circuit N2 exercis*") OR
AB (sprint N2 train*) OR
AB (sprint N2 exercis*) OR
AB (HIIT)

AND

TI (adher*) OR
TI (complian*) OR
TI (participat*) OR
TI (dropout) OR
TI (complet*) OR
TI (retention) OR
TI (attend*) OR
TI ("lack of uptake") OR
TI (frequen*) OR
TI ("lost to follow-up") OR
TI ("attendance frequen*") OR

AB (adher*) OR
AB (complian*) OR
AB (participat*) OR
AB (dropout) OR
AB (complet*) OR
AB (retention) OR
AB (attend*) OR
AB ("lack of uptake") OR
AB (frequen*) OR
AB ("lost to follow-up") OR
AB ("attendance frequen*") OR
MH (patient compliance+) OR
MH (patient dropouts) OR
MH (research subjects+) OR
MH (self-report+) OR
MH (questionnaires+) OR
MH (surveys)

Web of Science Legend:

- TS = search term is found within either the title, abstract, or keywords of an article.

TS=("high intensity" NEAR/3 train*) OR
TS=("high intensity" NEAR/3 exercis*) OR
TS=("aerobic interval train*") OR
TS=("aerobic circuit train*") OR
TS=("aerobic interval exercis*") OR
TS=("aerobic intermittent exercis*") OR
TS=("aerobic circuit exercis*") OR
TS=("interval train*") OR
TS=("intermittent train*") OR
TS=("circuit train*") OR
TS=("interval exercis*") OR
TS=("intermittent exercis*") OR
TS=("circuit NEAR/2 exercis*") OR
TS=(sprint NEAR/2 train*) OR
TS=(sprint NEAR/2 exercis*) OR
TS=(HIIT)

AND

TS=(adher*) OR
TS=(complian*) OR
TS=(participat*) OR
TS=(dropout) OR
TS=(complet*) OR
TS=(retention) OR
TS=(attend*) OR
TS=("lack of uptake") OR
TS=(frequen*) OR
TS=("attendance frequen*") OR
TS=("treatment adher*") OR
TS=("treatment complian*") OR
TS=("lost to follow-up") OR
